# Prognostic value of advanced imaging biomarkers for the progression of diabetic retinopathy: a systematic review

**DOI:** 10.1186/s13098-026-02159-3

**Published:** 2026-04-28

**Authors:** Salomão Antonio Oliveira, João Pedro R. Afonso, Rodrigo F. Oliveira, Carlos Hassel M. Silva, Deise A. A. P. Oliveira, Iransé Oliveira-Silva, Rodrigo A. C. Andraus, Rodolfo P. Vieira, Paolo Capodaglio, Alessandro R. Stival, Rafael Caiado C. Vencio, Sergio Vencio, Elias Jirjoss Ilias, Orlando A. Guedes, Claudia S. Oliveira, Wilson R. Freitas Júnior, Luís V. F. Oliveira, João Eduardo N. Salles

**Affiliations:** 1 Graduate Program in Health Sciences, Faculty of Medical Sciences of Santa Casa de São Paulo (PPGCS), São Paulo, 01224-001 SP Brazil; 2 Human Movement and Rehabilitation Graduate Program, Evangelical University of Goiás (UniEVANGÉLICA), Anápolis, 75083-515 GO Brazil; 3 Orthopaedic Rehabilitation Unit and Research Lab for Biomechanics, Rehabilitation and Ergonomics, Ospedale San Giuseppe, Istituto Auxologico Italiano, IRCCS, Piancavallo di Oggebbio, 28824 Italy; 4 Physical and Rehabilitation Medicine Sector, Department of Biomedical, Surgical and Dental Sciences, Università Degli Studi di Milano (UNIMI), Milan, 20122 Italy; 5 State Department of Health of Goiás, Goiânia, 74860-260 GO Brazil; 6 Reference Center in Ophthalmology (CEROF), Federal University of Goiás (UFG), Goiânia, 74605-020 GO Brazil; 7 ICF–Institute of Pharmaceutical Sciences, Aparecida de Goiania, Goiás, 74935- 530 Brazil; 8 Dentistry Graduate Program, Evangelical University of Goiás (UniEVANGÉLICA), Anápolis, 75083-515 GO Brazil

**Keywords:** Diabetic retinopathy, Retinal degeneration, Diabetes Mellitus, Proliferative diabetic retinopathy, Non-proliferative retinopathy

## Abstract

**Background:**

In recent years, diabetes mellitus (DM) has emerged as a chronic disease with a steadily increasing prevalence. It is closely associated with lifestyle and metabolic factors, including poor lifestyle habits that contribute to systemic metabolic alterations. Among its vascular complications, diabetic retinopathy (DR) is one of the most serious, with a high incidence rate and recognized as the leading cause of preventable blindness in working-age adults. One of the major challenges associated with DR is predicting disease progression. The transition from non-proliferative DR to proliferative DR is highly heterogeneous among individuals. This variability poses a substantial dilemma in clinical practice, as it hinders the routine identification of high-risk patients who require periodic monitoring and early intervention.

**Methods:**

This systematic review was conducted according to the guidelines of the Preferred Reporting Items for Systematic Reviews and was registered in the International Prospective Register of Systematic Reviews (CRD420251082364). This study included only longitudinal, prospective, and retrospective cohort studies published in any language. Eligible studies were required to report baseline markers of retinal neurodegeneration and DR status at the follow-up visit. A scientific literature search was conducted using PubMed, Scopus, and Web of Science, with no restrictions on publication dates. Additionally, a gray literature search was conducted in databases such as Google Scholar, OpenGrey, and ARVO (The Association for Research in Vision and Ophthalmology).

**Results:**

A total of 6,656 articles were retrieved from PubMed, Web of Science, and Scopus databases. After removing duplicates, 5,911 remained for title and abstract analyses. Of these, 33 studies were selected for full reading, of which 20 were excluded because they failed to meet the inclusion criteria, leaving 13 studies for systematic review. The certainty of evidence for the main identified predictors was rated as low for m-GCIPL thinning and very low for CC FD%, according to the GRADE approach.

**Conclusions:**

This systematic review demonstrates that advanced imaging biomarkers obtained using OCT and OCTA have shown consistent associations with DR progression. Markers quantifying vascular perfusion failure, such as CC FD% and neural structural damage, particularly m-GCIPL thinning, were identified as the most consistently reported and promising predictors of disease progression. While promising, these biomarkers currently present low to very low certainty of evidence, necessitating cautious clinical interpretation.

**Supplementary Information:**

The online version contains supplementary material available at 10.1186/s13098-026-02159-3.

## Introduction

 In recent years, diabetes mellitus (DM) has emerged as a chronic disease with a steadily increasing prevalence. It is closely associated with lifestyle and metabolic factors, including poor lifestyle habits that contribute to systemic metabolic alterations [1, 2]. Among its vascular complications, diabetic retinopathy (DR) is one of the most serious, with a high incidence rate and recognized as the leading cause of preventable blindness in working-age adults [3, 4]. DR progresses in two main clinical stages. The first stage, non-proliferative DR (NPDR), is characterized by vascular abnormalities. The second, more advanced stage, the proliferative DR (PDR), involves neovascularization and leads to a high risk of severe vision loss [5].

One of the major challenges associated with DR is predicting disease progression. The transition from NPDR to PDR is highly heterogeneous among individuals [6–8]. Although most patients with DM experience a certain degree of DR, only a small number progress to a severe state that affects vision [9]. This variability poses a substantial dilemma in clinical practice, as it hinders the routine identification of high-risk patients who require periodic monitoring and early intervention. Currently, all patients with DR require regular monitoring, which places a considerable burden on public health services. Consequently, patients who are not properly monitored are at an increased risk of vision loss [6, 9]. Therefore, predicting disease progression is crucial. Accurate prediction facilitates more effective management of healthcare resources, enabling high-risk patients to be consistently assessed and receive timely, appropriate treatment. This not only prevents vision loss but also optimizes the use of public health resources [10, 11].

The current gold standard for diagnosing and assessing DR progression is color fundus photography (CFR), which uses severity scales such as the Early Treatment Diabetic Retinopathy Study (ETDRS). This method allows for the assessment of disease severity and current status based on visible microvascular lesions [12–14]. However, this method has notable limitations, as it relies on pre-existing vascular lesions such as microaneurysms, hemorrhages, and exudates, which primarily reflect an early phase of the disease. Consequently, CFR assessment is largely limited to detecting subtle, early warning signs [11, 12], making it difficult to identify vascular changes that precede overt disease progression. DR is characterized by neurovascular degeneration, and it is well documented that retinal neuropathy can occur before vascular abnormalities are visible on fundus photography [15, 16].

Given the limitations of these methods, advanced imaging Technologies, such as optical coherence tomography (OCT), OCT angiography (OCTA), and ultra-wide-field angiography (UWFA), have emerged as valuable tools for early diagnosis and for generating evidence on the development of DR. These tools allow objective and noninvasive quantification of the structure of neural layers (OCT), perfusion of the retinal and choroidal microvasculature, and peripheral ischemia [17–19]. Recently, a growing number of longitudinal studies have reported the application of these tools to identify predictive biomarkers for DR [20–22]. Despite the potential of these technologies, findings remain scattered and derived from small cohorts, hindering the identification of robust biomarkers for routine clinical prognosis.

Given this gap, the objective of the present systematic review was to compile and critically analyze longitudinal studies evaluating advanced imaging biomarkers, such as OCT and OCTA, as independent predictors of DR progression. The goal was to provide a clear overview of the markers with the greatest promise for use as new clinical endpoints.

## Methods

This systematic review was conducted according to the guidelines of the Preferred Reporting Items for Systematic Reviews [23] and was registered in the International Prospective Register of Systematic Reviews (CRD420251082364).

### Eligibility criteria

This systematic review included only longitudinal, prospective, and retrospective cohort studies published in any language. Eligible studies were required to report baseline markers of retinal neurodegeneration and DR status at the follow-up visit. The minimum patient follow-up period was one year.

Regarding the population inclusion criteria, patients diagnosed with type 1 or type 2 DM were selected. Given that the focus of this review was disease progression, studies including patients across different baseline stages of diabetic retinopathy (including no DR, mild NPDR, and moderate NPDR) were considered eligible, provided that longitudinal progression outcomes could be assessed. We specifically looked for studies involving treatment-naive eyes or clarified if prior treatments like PRP or anti-VEGF were exclusion criteria to avoid confounding effects on retinal thickness and perfusion. The severity of DR was classified based on a standardized, validated scale, such as the ETDRS, to ensure homogeneity across the analyzed cohorts.

### Outcomes analyzed

The primary outcome was defined as clinically relevant progression of diabetic retinopathy, as reported in each included study. This included worsening on validated severity scales (e.g., ETDRS step progression), development of proliferative diabetic retinopathy, occurrence of diabetic macular edema, or other clinically significant endpoints such as referable diabetic retinopathy or treatment-requiring disease. Secondary outcomes included the association between baseline neurodegeneration markers and subsequent DR progression rate, and a qualitative analysis comparing the prognostic ability of different testing modalities evaluated in the studies.

### Search information

A scientific literature search was conducted using PubMed, Scopus, and Web of Science, with no restrictions on publication dates. The literature search was conducted up to July 2025. Additionally, a gray literature search was conducted using Google Scholar, OpenGrey, and the ARVO (The Association for Research in Vision and Ophthalmology) database. For Google Scholar, the first 200 results were screened. Relevant studies identified through gray literature sources were screened using the same eligibility criteria applied to indexed databases. Furthermore, the reference lists of all included studies were manually reviewed to identify additional eligible articles. Any records identified through these strategies were incorporated into the study selection process and evaluated independently by the reviewers.

### Search algorithms

The search strategy used in this review was as follows, with all keywords indexed as Medical Subject Headings (MeSH) terms:

Pubmed: ((“Diabetes Mellitus, Type 1“[Mesh] OR “Diabetes Mellitus, Type 2“[Mesh] OR “Diabetes Mellitus“[Mesh] OR “diabetic patient*“[Title/Abstract])) AND ((“Retinal Ganglion Cells“[Mesh] OR “Retinal Nerve Fibers“[Mesh] OR “Optical Coherence Tomography“[Mesh] OR “OCT Angiography“[Mesh] OR “Electroretinography“[Mesh] OR “Visual Field Tests“[Mesh] OR “retinal neurodegeneration“[Title/Abstract] OR “ganglion cell*“[Title/Abstract] OR “GCIPL“[Title/Abstract] OR “RNFL“[Title/Abstract] OR “OCT“[Title/Abstract] OR “OCTA“[Title/Abstract] OR “microperimetry“[Title/Abstract] OR “ERG“[Title/Abstract])) AND ((“Prognosis“[Mesh] OR “Incidence“[Mesh] OR “Disease Progression“[Mesh] OR “Longitudinal Studies“[Mesh] OR “Cohort Studies“[Mesh] OR “predictive value“[Title/Abstract] OR “prognostic“[Title/Abstract] OR “longitudinal“[Title/Abstract] OR “follow-up“[Title/Abstract] OR “progression“[Title/Abstract]))

Scopus: (TITLE-ABS-KEY (“Diabetes Mellitus, Type 1” OR “Diabetes Mellitus, Type 2” OR “Diabetes Mellitus” OR “diabetic patient*”)) AND (TITLE-ABS-KEY (“Retinal Ganglion Cells” OR “Retinal Nerve Fibers” OR “Optical Coherence Tomography” OR “OCT Angiography” OR Electroretinography OR “Visual Field Tests” OR “retinal neurodegeneration” OR “ganglion cell*” OR “GC-IPL” OR RNFL OR OCT OR OCTA OR microperimetry OR ERG)) AND (TITLE-ABS-KEY (Prognosis OR Incidence OR “Disease Progression” OR “Longitudinal Studies” OR “Cohort Studies” OR “predictive value” OR prognostic OR longitudinal OR “follow-up” OR progression)).

Web of Science: (TS=(“Diabetes Mellitus, Type 1” OR “Diabetes Mellitus, Type 2” OR “Diabetes Mellitus” OR “diabetic patient*”)) AND (TS=(“Retinal Ganglion Cells” OR “Retinal Nerve Fibers” OR “Optical Coherence Tomography” OR “OCT Angiography” OR Electroretinography OR “Visual Field Tests” OR “retinal neurodegeneration” OR “ganglion cell*” OR “GC-IPL” OR RNFL OR OCT OR OCTA OR microperimetry OR ERG)) AND (TS=(Prognosis OR Incidence OR “Disease Progression” OR “Longitudinal Studies” OR “Cohort Studies” OR “predictive value” OR prognostic OR longitudinal OR “follow-up” OR progression)).

### Study selection

The study selection process followed a two-phase approach managed via Rayyan software [24]. To account for variations in terminology across imaging platforms (e.g., GC-IPL vs. GCIPL) and minimize the impact of restrictive Boolean logic, two reviewers (SAO and JPRA) independently screened all retrieved titles and abstracts. This phase specifically targeted synonyms for retinal neurodegeneration and various predictive markers to ensure that relevant studies were not excluded due to metadata inconsistencies. In the second phase, the same reviewers independently performed a full-text review to confirm eligibility. Any disagreements throughout the process were resolved through discussion with a third reviewer (LVFO).

### Data extraction

The data extraction process was performed independently by two reviewers using a standardized, predefined form in accordance with the PRISMA recommendations. To ensure consistency in data collection, the form was first tested by both reviewers on a small sample of studies before initiating the full extraction. Any discrepancies that arose during the study were resolved by a third reviewer. The following information was extracted from each study: identifying data, including author, year of publication, and country; methodological details, such as study design, duration of follow-up; population characteristics, including demographics (sample size, age, sex) and clinical data (type and duration of DM, initial glycemic control); methods used to assess neurodegeneration, DR status at baseline and of follow-up; and measures of prognostic association, such as hazard ratios (HRs) and odds ratios (ORs), with their respective 95% confidence intervals (CIs).

### Data synthesis

Initial quantitative synthesis was attempted; however, due to significant methodological heterogeneity (I^2^ = 96.0% for CC FD%), inconsistent reporting of effect measures (OR, RR, and HR), and variations in imaging platforms, a formal meta-analysis was deemed inappropriate. Instead, a robust systematic narrative synthesis was performed to avoid biased estimates and ensure a reliable overview of the current evidence.

### Risk of bias

For the included studies, the methodological quality and risk of bias were independently assessed by two reviewers using the QUIPS (Quality in Prognosis Studies) tool [25]. This tool assesses the validity of prognostic factor studies and structures the analysis into six main domains that are considered the most important sources of bias in this type of study: study Participation, which assesses sample representativeness; study attention, which analyzes the impact of losses to follow-up; prognostic factor measurement; outcome measurement; study confounding, which examines the control of confounding variables; and statistical analysis and reporting. The risk of bias was assessed and classified as low, moderate, or high for each domain. Disagreements between reviewers were resolved by consensus or mediation by a third reviewer.

The results of this assessment were used in two distinct ways: first, for quality description, where the findings were summarized and presented to illustrate the overall methodological quality of the available evidence.; and second, for interpretation and certainty of evidence, where study quality served as a key factor in the final interpretation of the results and was incorporated into the assessment of the certainty of the evidence for each outcome.

### Certainty of evidence assessment (GRADE)

The overall certainty of the evidence for each primary outcome was assessed by two independent reviewers using the GRADE (Grading of Recommendations, Assessment, Development, and Evaluation) approach [26]. Evidence was classified as high, moderate, low, or very low based on domains such as risk of bias, inconsistency, indirectness, imprecision, and publication bias.

## Results

### Study selection

**Fig. 1:**
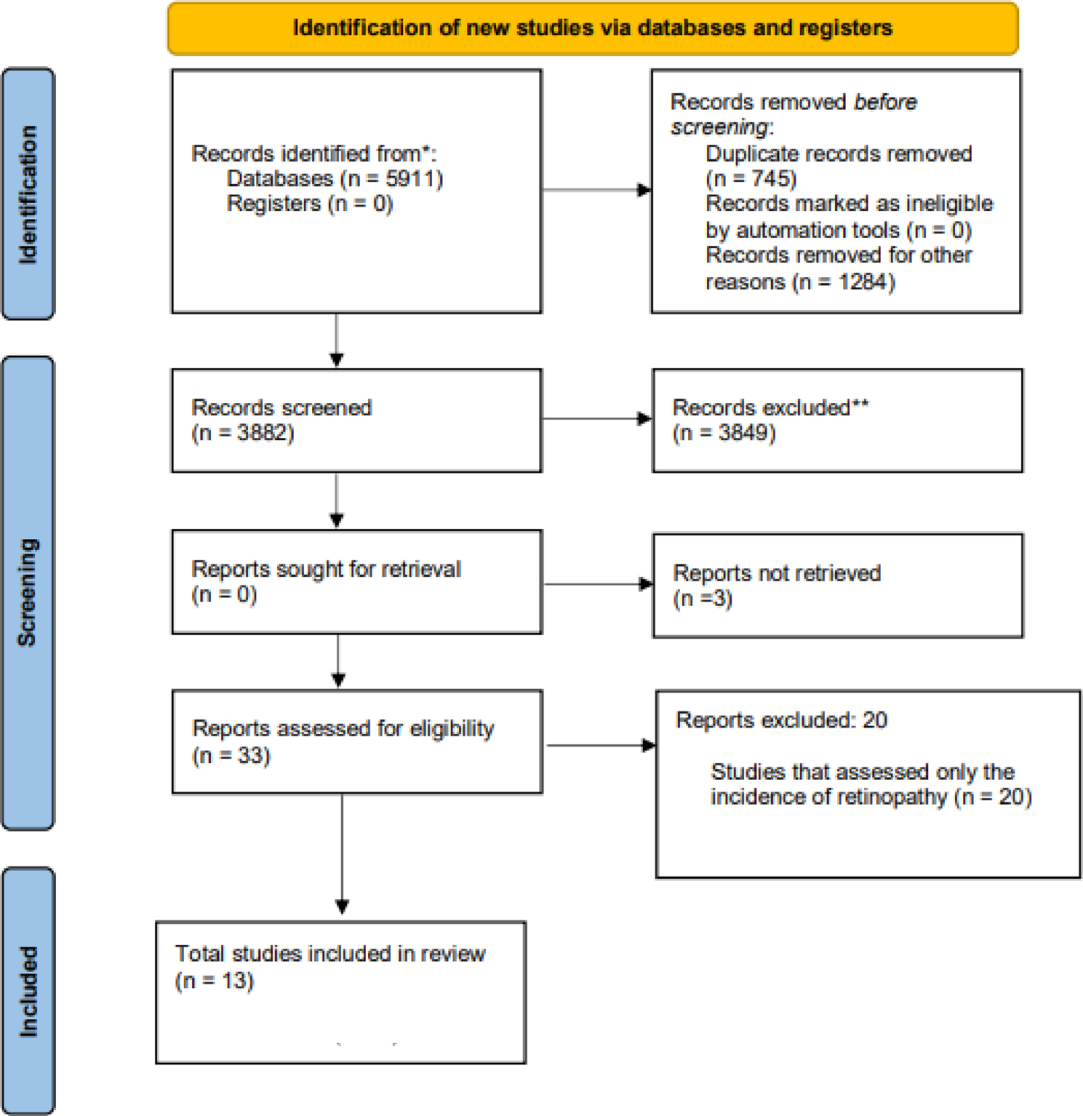
Flow diagram of the current systematic review conducted according to the Preferred Reporting Items for Systematic Reviews and Meta-analysis (PRISMA) guidelines. *Data sources: PubMed, Scopus and Web of Science; search conducted until July 2025. **Records excluded during initial screening by title and abstract (*n* = 3849) for not meeting the pre-defined inclusion criteria for the research question

A total of 6,656 articles were retrieved from PubMed, Web of Science, and Scopus databases. After removing duplicates, 5,911 remained for title and abstract analyses. Of these, 33 studies were selected for full reading, of which 20 were excluded because they failed to meet the inclusion criteria, leaving 13 studies for systematic review (Fig. 1).

### Characteristics of studies

Regarding the study locations, most were conducted in Asia: 8 in China, 1 in Azerbaijan, 1 in South Korea, 1 in India, 1 in the United States, and 1 in Austria. Of these 13 studies, 10 were prospective cohort studies and 3 were retrospective cohort studies. Additional study characteristics are presented in Tables 1 and 2.

**Table 1:** Characteristics of included studies

Source (Year)	Location	Study design	Age (baseline)	Duration of diabetes (years)	Final sample	Main outcomes	Assessment/classification of DR (baseline)
Srinivasan et al. (2023) [27]	Índia	Prospective cohort	Mean: 58.4 years	Mean: 11.3 years	206 eyes	Progression: 37.7% (17/45)	Ultra-widefield photography. 161 eyes without DR; 45 with NPDR
Gong et al. (2022) [28]	China	Prospective cohort	Mean: 65.0 years	Mean: 9.2 years	895 patients	Progression: 7.5% (11/147)	Seven-field photography (ETDRS). 748 without DR; 147 with NPDR (28 mild, 119 moderate)
Wu et al. (2023) [29]	China	Prospective cohort	Mean: 64.4 years	Mean: 13.2 years	233 eyes (125 patients)	Progression 17.2% (40 eyes)	Seven-field photography (ETDRS). All with mild or moderate NPDR
Chen et al. (2022) [30]	China	Prospective cohort	Mean: ~64.0 years	Mean: 8.4–10.4 years	1805 eyes (903 patients)	Progression of DR: 16.3%. Development of DME: 6.5%.	Seven-field photography (ETDRS). All without DR or with mild NPDR
Liang et al. (2025) [31]	China	Prospective cohort	Mean: 63.8 years	Mean: 11.3 years	182 patients (progression)	Progression: 22.0% (40/182).	Seven-field photography (ETDRS). All without DR, or with mild/moderate NPDR
Majidova (2024) [32]	Azerbaijan	Retrospective cohort	Mean: 56.0–57.5.0.5 years	Mean: 12.0–13.5.0.5 years	82 patients	Progression from NPDR to PDR: 51.2% (42/82)	Seven-field photography (ETDRS). All with moderate NPDR at initial evaluation
Aschauer et al. (2020) [33]	Austria	Prospective cohort	Mean: 57 years	Mean: 11 years	117 eyes (59 patients)	Progression: 11.1% (13 eyes).	Fundoscopy (ICDR). 105 without DR, 6 mild NPDR, 6 moderate NPDR
Kim et al. (2018) [34]	South Korea	Retrospective cohort	Mean: 64.1 years	Mean: 10.6–15.5 years	87 eyes	Two-step progression: 44.8%. Progression to PDR: 6.9%	Seven-field photography (ETDRS). 28.8% without DR; 71.2% with mild NPDR
Wang et al. (2022) [35]	China	Prospective cohort	Mean: 63.8 years	Mean: 8.4 years	1.345 patients	DR progression 36,9%	Seven-field photography (ETDRS). 95.2% without DR; 4.8% with mild NPDR
Guo et al. (2022) [36]	China	Prospective cohort	Mean: 64.1 years	Mean: 8.5 years	1879 eyes (946 patients)	DR progression: 16.6%. DME development: 6.1%.	Seven-field photography (ETDRS). All without DR or with mild NPDR
Yuan et al. (2022) [37]	China	Prospective cohort	Mean: 63.8–65.0 years	Mean: 8.7–9.4 years	1.033 patients	Progression: 7.4%.	Seven-field photography (ETDRS). 886 without DR; 147 with DR
Ding et al. (2024) [38]	USA	Retrospective cohort	Median: 64.0 years	Mean: 15.8 years	88 eyes (57 patients)	Significant clinical outcomes: 19.3% (includes progression, DME, and treatment)	Ultra-widefield photography. All with NPDR (29 mild, 46 moderate, 13 severe)
Tang et al. (2025) [39]	China	Prospective cohort	Mean: 60.0 years	Mean: 12.2 years	385 eyes (215 patients)	Progression: 25.7%. Development of PDR: 9.9%	Fundus photography (ETDRS). 178 without DR; 207 with NPDR

DR stands for Diabetic Retinopathy, which is classified into Non-Proliferative Diabetic Retinopathy (NPDR) and Proliferative Diabetic Retinopathy (PDR). A related complication is Diabetic Macular Edema (DME). The severity of DR is graded using standardized scales, such as the Early Treatment of Diabetic Retinopathy Study (ETDRS) scale and the International Clinical Diabetic Retinopathy (ICDR) scale

**Table 2:** Summary of imaging biomarkers with prognostic value for disease progression

Author, Year	Modality	Parameter and measurement method	Baseline Value (Group with Outcome)	Baseline Value (Group without Outcome)	Prognostic Factor (Multivariate Analysis)	Parameter Specific Notes
Srinivasan, 2023 [27]	OCTA	Macular Vascular Density (VD) (mm/mm^2^)	12.90 ± 3.22%	14.90 ± 2.28%	HR = 0.825 (IC 95%: 0.731–0.931; *p* = 0.002)	The risk of progression increases 1.21-fold for each unit decrease in vascular density
Srinivasan, 2023 [27]	OCTA	Macular perfusion Macular Perfusion (MP) (%)	31.79 ± 8.76	36.96 ± 6.16	HR = 0.936 (IC 95%: 0.892–0.982; *p* = 0.007)	The risk of progression increases 1.10-fold for each unit decrease in macular perfusion
Gong, 2022 [28]	OCT	Mean RNFL thickness (µm)	111.6 ± 13.8 μm	109.7 ± 13.7 μm	RR = 1.17 (IC 95%: 0.60–2.27; *p* = 0.648)	Mean pRNFL thickness was not a statistically significant predictor of DR progression. The study uses Risk Ratio (RR)
Gong, 2022 [28]	OCT	pRNFL thickness in the inferior quadrant (µm)	146.5 ± 27.9 μm	142.7 ± 24.3 μm	RR = 1.23 (IC 95%: 0.59–2.57; *p* = 0.577)	Lower quadrant pRNFL thickness was not a statistically significant predictor of DR progression
Wu, 2022 [29]	OCTA	Fractal Dimension (FD) in DCP	1.8560 ± 0.0271	1.8660 ± 0.0078	OR = 2.484 (IC 95%: 1.268–4.867; *p* = 0.008)	A lower fractal dimension (less complex network) in DCP was associated with progression. The OR is per standard deviation decrease
Wu, 2022 [29]	OCTA	Vascular Tortuosity (BVT) in DCP	1.0067 ± 0.0020	1.0057 ± 0.0008	OR = 2.076 (IC 95%: 1.382–3.121; *p* < 0.001)	Greater tortuosity in DCP was associated with progression. The OR is per standard deviation increase
Chen, 2022 [30]	SS-OCTA	Mean CC FD (%): Percentage of flow deficit in the choriocapillaris in a 3 × 3 mm macular area	27.93 (2.03) %	25.02 (3.67) %	OR = 3.41 (IC 95%: 2.65–4.39; *p* < 0.001)	The Odds Ratio (OR) is per 1 standard deviation increase in CC FD%. A greater flow deficit (higher CC FD%) increased the risk of DR progression
Liang, 2024 [31]	SD-OCT	Interdigitation Zone Length IZ Length, mm): Objectively measured from Longitudinal Reflectivity Profiles (LRPs)	3.758 ± 1.653 mm	5.722 ± 0.865 mm	OR = 0.039 (IC 95%: 0.011 − 0.139; *P*<0.001)	A longer IZ length is associated with a lower risk (protective effect) of DR progression at 3 years. The OR is per 1 mm increase in IZ length. The combination of IZ with risk factors achieved an AUC of 0.953 in predicting progression
Majidova, 2024 [32]	SS-OCTA	Serum Analysis (ELISA) + ERG	EPO in Group II: 35.2 ± 2.2 mIU/mL	EPO in Group I: 23.7 ± 1.7 mIU/mL	High-risk cutoff: 27.5 mIU/mL	An EPO level exceeding 27.5 mIU/mL predicts a high risk of progression from NPDR to PDR. The baseline EPO level was already significantly higher in Group II
Aschauer, 2024 [33]	OCTA and SD-OCT	SVC Vessel Density (SVC VD, %/year): Measurement of the area of ​​blood flow in the Superficial Capillary Plexus (6 × 6 mm)	27.2 ± 3.1%	28.5 ± 2.6	Not Calculated/Not Applicable.	The reduction in VD SVC and thinning of the GCL/IPL layer occurred in parallel and are highly progressive
Kim, 2018 [34]	OCT	mGCIPL thinning rate (µm/year): Rate of thickness loss of the ganglion cell layer and inner plexiform layer in the macula	0.39 ± 0.31 μm/year.	0.22 ± 0.30 μm/year	HR = 1.924 (IC 95%: 1.026–3.606; *p* = 0.041)	The rate of mGCIPL thinning was an independent predictor for DR progression
Wang, 2023 [35]	SS-OCT	Average Choroidal Thickness: Automatic average of choroidal thickness across the nine ETDRS grid fields 1, measured by swept-source optical coherence tomography (SS-OCT)	166.3 ± 55.6 μm	199.3 ± 62.5 μm	RR = 0.903 (95% CI: 0.871–0.935; *P* < 0.001)	The Relative Risk (RR) is for every 10-µm increase in average choroidal thickness. An RR of 0.903 indicates a protective effect, meaning that a thinner choroid is associated with a higher risk of RDR progression
Guo, 2024 [36]	SS-OCTA	CC Flow Deficit Percentage (CC FD%, %): Percentage of flow deficit in the peripapillary choroidal capillary plexus (choriocapillaris) (3 × 3 mm)	CC FD% (Total Cohort): 29.57 ± 5.48%	CC FD% (Total Cohort): 26.67 ± 6.98%	RR = 1.62 (IC 95%: 1.40 − 1.88; *P*<0.001)	The RR is for every 1 standard deviation (SD) increase in CC FD%. A high CC FD% indicates a 62% increased risk of DR progression. CC FD% adds significant predictive value to traditional factors
Yuan, 2023 [37]	SS-OCTA	Whole-image Peripapillary Vessel Length Density (wi-pVLD, por 1-SD increase)	Mean CC FD% (Total Cohort): 17.92 ± 2.45 mm^− 1^	Mean CC FD% (Total Cohort): 18.71 ± 2.22 mm^− 1^	RR = 0.46 (IC 95%: 0.33 − 0.66; *P*<0.001)	The RR is for every 1 standard deviation (SD) increase in wi-pVLD. An RR of 0.46 indicates that a higher wi-pVLD is associated with a 54% reduction in the risk of RDR
Ding, 2024 [38]	SS-OCTA	Ischemia Index (ISI): Ratio of Non-Perfusion Area (NPA) to the total analysis area (12 × 12 mm)	SI ≥ 0.073 (Highest risk group - Group 3) Baseline ISI for the entire cohort (median): 0.03.	ISI ≤ 0.038 (Lowest risk group - Group 1)	HR = 1.05 (IC 95%: 1.02 − 1.09; *P*=0.004)	HR is for each unit increase in ISI. A higher ISI is associated with higher risk. Another strong predictor was Unstable IRMAs (HR 3.88)
Tang, 2024 [39]	OCT	m-GCIPL Mean (µm): Average thickness of the GCIPL layer in the macular area	81.0 ± 5.9 μm	83.6 ± 6.0 μm.	HR = 1.516 (IC 95%: 1.243 − 1.850; *P*<0.001)	The HR is for every 1 standard deviation (SD) reduction in m-GCIPL thickness. A thinner m-GCIPL is associated with a 51.6% higher risk of DR progression

Imaging modalities: OCT (Optical Coherence Tomography), OCTA (Optical Coherence Tomography Angiography), SS-OCT (Swept-Source Optical Coherence Tomography), SS-OCTA (Swept-Source OCT Angiography), SD-OCT (Spectral-Domain OCT) and EFOV (Expanded-Field of View OCTA). Biomarker Parameters: Structural/Neural: m-GCIPL (Macular Ganglion Cell-Inner Plexiform Layer), IZ (Interdigitation Zone), and pRNFL (Peripapillary Retinal Nerve Fiber Layer). Vascular/Perfusion: ISI (Ischemia Index), CC FD% (Choriocapillaris Flow Deficit Percentage), DCP (Deep Capillary Plexus), FD (Fractal Dimension), SVC (Superficial Vascular Complex), VD (Vessel Density), VT (Vascular Tortuosity, also as BVT: Branching Vascular Tortuosity), and wi-pVLD (Whole-image Peripapillary Vessel Length Density). Other Parameters: CT (Choroid+++++al Thickness) and EPO (Erythropoietin, measured by Serum Analysis)

### Characteristics of study populations

The 13 studies included in this analysis encompassed broad and diverse populations of patients with diabetes. The average age of participants at baseline ranged from 56 to 65 years. The average duration of diabetes also varied across studies, ranging from approximately 8.4 to 15.8 years.

### Retinopathy status at baseline

The included studies evaluated patients across a spectrum of baseline diabetic retinopathy stages, ranging from no DR to mild and moderate NPDR, depending on the study design. Retinopathy severity was primarily classified through fundus photography using standardized scales such as the ETDRS.

### Risk of bias

**Fig. 2:**
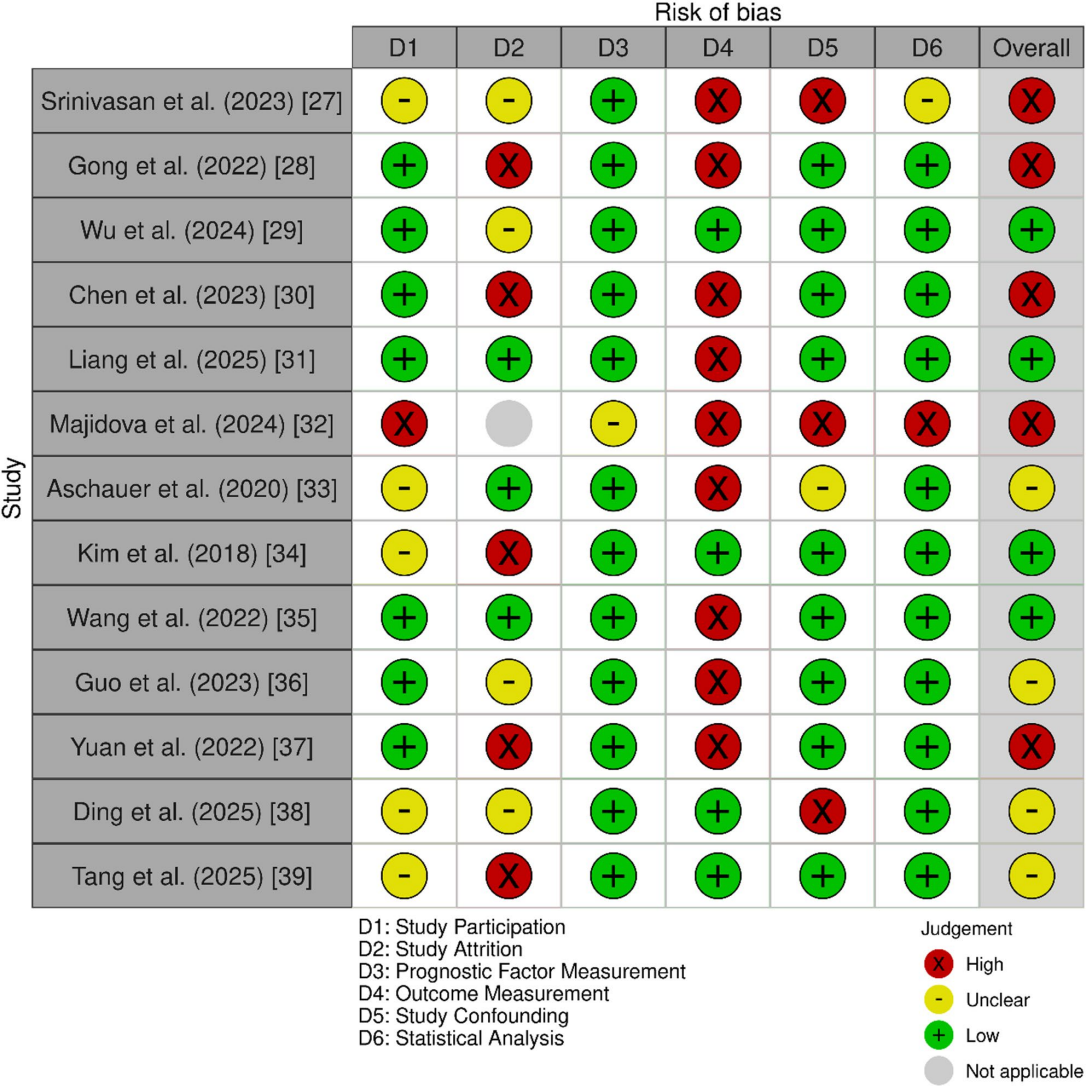
Summary of Risk of Bias (Bias Plot) for the included studies using the QUIPS tool. The assessment was performed for each study (rows) across six bias domains (D1 to D6), as defined by the QUIPS tool. The colors indicate the risk of bias judgment for each domain: Green (Low Risk), Yellow (Unclear Risk), and Red (High Risk). The “Overall” column represents the overall Risk of Bias for each study

The risk of bias and methodological quality of the studies included in this review were assessed using the QUIPS tool. Of the 13 studies, five (38%) were classified as having a high risk of bias [27–31], four (31%) as having a moderate risk [32–35], and four (31%) as having a low risk [32, 36–38]. The detailed assessment of each domain is presented in Fig. 2.

Domain analysis revealed that the most critically biased items were related to outcome measurements and study attention. Most studies were classified as having a high risk of bias, while others were classified as having a moderate or low risk of bias [27–32], 38– [31]. However, the studies did not describe whether the evaluators who classified retinopathy progression were blinded to the prognostic factor data, introducing a high risk of detection bias. Furthermore, attrition bias was observed in several studies [28, 29, 31, 35, 39], with attrition rates exceeding 20%, ​​or systematic differences observed between participants who lost to follow-up and those who completed follow-up.

Confounding bias was noted in some studies [27, 30, 34], that did not adjust for key confounders such as glycemic control (HbA1c) and baseline retinopathy severity. Conversely, most studies exhibited a low risk of bias in the prognostic factor measurement domains owing to the use of standardized and objective imaging methods and statistical tests, as well as the application of appropriate multivariate regression models.

### Structural biomarkers of neurodegeneration

Analysis of the included studies revealed that several structural changes in the retina, as measured using OCT, have prognostic value in the progression of DR. The main biomarkers evaluated were the thicknesses of the ganglion cell and inner plexiform layer (GCIPL), peripapillary retinal nerve fiber layer (pRNFL), and length of the interdigitation zone (IZ).

### Ganglion cell and inner plexiform layer

Two included studies evaluated the prognostic potential of the macular GCIPL (m-GCIPL) in the progression of DR [35, 39], indicating that both established degeneration and its rate of progression are risk factors. The rate of m-GCIPL thinning was identified as an independent predictor for DR progression, with the risk of progression increasing almost twofold for each micrometer per year of thinning (HR = 1.924; 95% CI: 1.026–3.606; *p* = 0.041) [39]. Regarding baseline thickness, a lower mean m-GCIPL thickness at the beginning of the study was associated with a higher risk of disease progression (HR = 1.516; 95% CI: 1.243–1.850; *p* < 0.001). Each one standard deviation (SD) decreases in m-GCIPL thickness increased the risk of progression by 51.6% [35].

### Peripapillary retinal nerve fiber layer

One study assessed the thickness of the pRNFL as a potential biomarker. However, in this longitudinal study, neither the mean pRNFL thickness (relative risk [RR] = 1.17; *p* = 0.648) nor the inferior quadrant-specific thickness (RR = 1.23; *p* = 0.577) was a statistically significant predictor of DR progression [28].

### Interdigitation zone

One study analyzed the prognostic value of IZ length, as it is associated with photoreceptor health [37]. A greater baseline IZ length was strongly associated with a lower risk of DR progression at 3 years, indicating a significant protective effect. The OR was 0.039 (95% CI: 0.011–0.139; *p* < 0.001) for each 1-mm increase in IZ length. The authors highlighted that combining this measurement with other risk factors yielded excellent predictive capacity (area under the curve [AUC] = 0.953) [37].

### Vascular and perfusion biomarkers (OCTA and swept-source [SS]-OCTA)

Changes in the microvasculature of the retina and choroid, as assessed by OCTA, are crucial predictors of DR progression. The included studies categorized these findings into three main biomarker groups: vascular density (VD) and perfusion; vascular network geometry; and flow deficits and ischemia.

Regarding the measurement of vascular metrics, considerable variation was observed in image processing techniques. For the percentage of cortical flow deficit (CC FD%), studies primarily used a 3 × 3 mm macular scan area. However, the software and algorithms for quantifying flow deficits varied between source-scanning OCT (SS-OCTA) and spectral-domain OCT (SD-OCT) platforms. This technical heterogeneity in image binarization and thresholding methods across studies is a factor that may influence the consistency of the reported predictive values.

### Vascular density and perfusion

Multiple studies have investigated vascular network density as a prognostic factor for different anatomical locations. Analyses of the macula revealed that disease progression was associated with lower VD and lower macular perfusion (MP). For each unit decrease in VD, the risk of progression increased 1.21-fold (HR = 0.825; *p* = 0.002), and for each unit decrease in MP, the risk increased 1.10-fold (HR = 0.936; *p* = 0.007) [27]. A similar finding was observed in another study, where a highly progressive reduction in vessel density in the superficial capillary plexus (SVC) occurred in parallel with neural thinning, although the study did not calculate a specific multivariate risk factor for this parameter [32]. Conversely, in the peripapillary region, a higher whole image peripapillary vessel length density was associated with a protective effect, reducing the risk of developing DR by 54% (RR = 0.46) [31]. Collectively, these findings suggest that the rarefaction of the microvasculature, especially the macula, is a risk factor for DR progression.

### Vascular network geometry

Geometric characteristics of the vasculature also possess prognostic value. Analysis of the deep capillary plexus revealed that a smaller fractal dimension and greater vascular tortuosity were both associated with a significantly higher risk of disease progression (OR = 2.484 and OR = 2.076, respectively) [36].

### Flow deficits and ischemia

Measures that quantify vascular perfusion failure have been identified as the most consistently reported and promising predictors of DR progression. Two independent studies demonstrated that a higher percentage of choriocapillaris flow deficit (CC FD%) significantly increases the risk of DR progression [29, 33]. Chen et al. reported an OR of 3.41, while Guo et al. reported a RR of 1.62 for every 1 SD increase in CC FD%. Consistently, another study using a broader metric of nonperfusion, the ischemic index (ISI), also demonstrated that a higher ISI was associated with a greater risk of adverse clinical outcomes, including DR progression (HR = 1.05) [34]. These three studies, which used complementary metrics, provided strong evidence that hypoperfusion in the choroid and retina is a key prognostic factor for DR progression.

### Other prognostic markers

In addition to neurodegenerative and vascular perfusion biomarkers, other imaging and systemic parameters have demonstrated prognostic value. Choroidal thickness, measured using SS-OCT, is an important structural biomarker [38]. The authors concluded that a thinner choroid was an independent risk factor for developing referrable DR (RDR) at 2 years. Patients who progressed to RDR had a significantly lower mean choroidal thickness at baseline (166.3 μm) compared with those who remained stable (199.3 μm). The risk of RDR increased as the choroid became thinner (RR = 0.903 for every 10 μm increase in thickness). Notably, the addition of choroidal thickness measurements to conventional risk factors (such as HbA1c and blood pressure) significantly improved the predictive ability of the model, increasing the AUC from 0.708 to 0.761 [38].

Using distinct and diverse methods, combining serum (ELISA), functional (electroretinogram [ERG]), and imaging (SS-OCTA) analyses, serum erythropoietin (EPO), a systemic marker, was identified as a high-risk predictor of progression from NPDR to PDR [30]. A cut-off point of 27.5 mIU/mL was established, above which the risk of progression was considered high. The group that progressed had significantly higher EPO levels at baseline (35.2 ± 2.2 mIU/mL) compared with the group that did not experience disease progression (23.7 ± 1.7 mIU/mL). This finding highlights the potential of systemic biomarkers to reflect the body’s response to hypoxia and predict disease progression [30].

### Analysis of secondary outcomes

#### Association with speed and degree of DR progression

Analysis of the included studies revealed that certain biomarkers could not only predict DR progression but also provide insights into its dynamics and speed. Notably, the most striking finding was the rate of thinning of the m-GCIPL over time [39]. Using a measurement of speed (µm/year), a faster rate of thinning was identified as an independent predictor of DR progression, suggesting that the rate of neurodegeneration is directly linked to the rate of clinical disease progression. Supporting this idea, the reduced VD in the superficial plexus and thinning of the GCL/IPL were highly progressive processes that occurred in parallel, indicating that the dynamics of these changes were associated with a more aggressive disease course [32]. Two other studies showed that a higher degree of initial neurodegeneration (thinner m-GCIPL) or greater vascular rarefaction at baseline conferred a significantly higher risk of progression [27, 35]. This reinforces the hypothesis that more severe neurovascular damage at baseline may be indicative of a more advanced disease that tends to progress rapidly to the advanced stages.

### Qualitative comparison of prognostic ability between modalities

#### Biomarkers of vascular perfusion

Biomarkers that directly quantify vascular perfusion failure appear to be the most consistent and robust predictors of disease progression. CC FD% emerged as a particularly robust marker, with two independent studies reporting that a higher CC FD% substantially increases the risk of DR progression [29, 33]. The consistency of findings across these two cohort studies strengthens this evidence. Supporting this argument, the ISI, which also measures the area of ​​nonperfusion, was identified as a key predictor of adverse clinical outcomes. The strength of these markers lies in their ability to directly measure ischemia, which is a central event in the pathophysiology of DR.

#### Biomarkers of structural neurodegeneration

Biomarkers that measure structural damage to the neural layers of the retina, particularly the m-GCIPL, were also identified as interesting predictors. Both the thickness of the m-GCIPL at baseline and its rate of thinning over time were independently associated with DR progression [35, 39], suggesting that m-GCIPL may serve as an indicator of both cumulative damage and disease progression rate. Notably, the IZ length exerts a notable protective effect, indicating that photoreceptor health plays a crucial prognostic role [37].

#### Biomarkers with limited or inconsistent prognostic value

Contrary to the robust evidence supporting the prognostic value of the m-GCIPL, pRNFL thickness alone demonstrated limited predictive utility across the included studies. One study found no statistically significant association between pRNFL thickness and DR progression in multivariate analysis [28], indicating that neurodegenerative damage associated with DR progression manifests more prominently and earlier in the m-GCIPL than in the peripapillary region.

### Certainty of evidence (GRADE)

The overall certainty of evidence for the main identified prognostic biomarkers was assessed using the GRADE approach. For the choriocapillaris flow deficit (CC FD%), the certainty of evidence as a predictor of DR progression was rated as very low. This downgrade was due to serious limitations in the risk of bias, very serious inconsistency between individual study results, and imprecision. Regarding m-GCIPL damage as a predictor, the certainty of evidence was classified as low. This classification resulted from serious limitations in the risk of bias and serious indirectness, as the included studies employed different measurement methods, such as thinning rates versus baseline thickness. In summary, while this systematic review indicates significant independent associations, the low to very low certainty of evidence necessitates a cautious interpretation of these biomarkers’ predictive power in routine clinical settings.

## Discussion

This systematic review synthesized longitudinal evidence on the prognostic value of advanced imaging biomarkers for DR progression. Vascular hypoperfusion, quantified by the CC FD%, and structural neural damage in the macula, reflected by thinning of the m-GCIPL, emerged as the most consistently reported and promising predictors of DR progression across the included studies [29, 33, 35, 39]. In contrast, evidence supporting the prognostic value of pRNFL thickness was limited and did not demonstrate a significant association with DR progression in multivariate analysis [28]. Other biomarkers, such as IZ length and choroidal thickness, also demonstrated potential prognostic value [37, 38].

### Biological and clinical interpretation

The findings of this review clarify that DR should be considered a complex neurovascular disease. Recent studies have further emphasized the contribution of molecular and metabolic pathways in retinal injury, supporting the concept of diabetic retinopathy as a multifactorial neurovascular disorder [40]. The strong prognostic association observed for CC FD%, supported by the findings related to the ISI, highlights the central role of hypoperfusion and ischemia as key accelerators of disease progression [29, 33, 34]. These longitudinal data corroborate the hypothesis that alterations in the microvasculature of the choroid and retina, detectable by OCT, may precede microvascular lesions visible on fundus photography, thereby providing an earlier window for risk identification.

Another valuable finding was the discovery of the importance of location in assessing neurodegeneration. Although neural damage in the macula (thinning of the m-GCIPL) showed strong prognostic value, the same was not observed in the pRNFL [28, 35, 39]. Accordingly, the macula, with its high metabolic demand, may be more vulnerable to early diabetic damage, whereas peripapillary measurements, reflecting axons throughout the retina, could dilute the signal of early macular damage. These findings strongly indicate the need to focus on the prognostic assessment of the macular region. Conceptual frameworks from other ophthalmic conditions, such as glaucoma, further support the development of structured biomarker-based diagnostic and prognostic models, highlighting the potential for cross-disciplinary integration in retinal diseases [41].

### Geographic bias

A significant geographical bias was observed, as 8 out of the 13 included studies were conducted in China. This concentration limits the generalizability of our findings to other ethnicities, as racial variations in retinal vascular density and neural thickness may influence the prognostic baseline of these biomarkers.

### Heterogeneity of devices

Technical heterogeneity remains a major challenge. Differences between Spectral-Domain (SD-OCT) and Swept-Source (SS-OCT) platforms, along with proprietary binarization algorithms for quantifying flow deficits (CC FD%), introduce significant variability. These discrepancies hinder the direct comparison of absolute values across different clinical settings.

### Clinical cut-offs

Despite the prognostic potential of CC FD% and m-GCIPL, universal clinical cut-off values have not yet been established. While some studies suggested high-risk thresholds for systemic markers like EPO (27.5 mIU/mL)^30^, further longitudinal research is mandatory to validate specific imaging thresholds that can reliably guide routine clinical decisions. This necessity is reinforced by the current technical heterogeneity between OCT platforms, which hinders the standardization of diagnostic cut-offs for vascular and neural metrics.

### General strengths and limitations

This review was conducted using tools that markedly increased the methodological rigor of the research. The protocol was registered with PROSPERO and followed the PRISMA guidelines. Furthermore, the risk of bias in the included studies was assessed using the QUIPS tool, and the GRADE system was used to analyze the certainty of evidence.

Nevertheless, some limitations in the included studies may have affected the results of this review. A major concern was the high risk of bias in the studies included, with 69% classified as moderate to high risk, primarily due to the lack of evaluator blinding and, in some cases, high rates of loss to follow up. Additionally, heterogeneity in imaging methods, definitions of disease progression, characteristics of study populations (a notable predominance of Asian cohorts), and follow-up duration limits the generalizability of the findings and the possibility of direct comparisons between studies.

Another important limitation is the heterogeneity in outcome definitions across the included studies. Different studies evaluated distinct clinical endpoints, including ETDRS step progression, development of proliferative diabetic retinopathy, diabetic macular edema, referable diabetic retinopathy, and composite outcomes. These endpoints represent different pathophysiological processes and should not be interpreted as equivalent, which may influence the comparability and interpretation of the reported prognostic associations.

### Implications for clinical practice and future research

Considering the low certainty of evidence, imaging biomarkers evaluated in this review, while promising, are not currently suitable for routine implementation in the risk stratification of DR progression in clinical practice. However, the consistent findings for CC FD% and m-GCIPL suggest that they may, in select cases and as complementary tools, help identify patients who could benefit from more frequent or intensive monitoring. In this context, the integration of prognostic biomarkers with emerging therapeutic strategies may contribute to more personalized management approaches in diabetic retinopathy [42].

While statistically significant, the biomarkers identified (CC FD% and m-GCIPL) should be interpreted with caution due to the current low certainty of evidence. Furthermore, this review focused on anatomical progression (ETDRS scale). Future studies must correlate these imaging changes with patient-centered outcomes, such as visual acuity and contrast sensitivity, to establish true clinical utility.

The main implication of this systematic review is the evident need for additional longitudinal studies with follow-up periods aligned with methodological standards, such as those recommended by PRISMA and GRADE, to enhance study design and the quality of evidence. Future studies should prioritize larger, more ethnically diverse samples, follow up patients for a prolonged period, and, most importantly, rigorously standardize imaging protocols, specific biomarker definitions, and disease progression criteria. Validating clinically useful cutoffs for CC FD% and m-GCIPL is essential, as this will enable better comparisons in the search for more concrete evidence.

Ultimately, randomized clinical trials are required to determine whether management strategies guided by these biomarkers can alter the natural disease course and prevent vision loss in patients with diabetes.

In summary, while our results point to a promising prognostic role for these imaging biomarkers, the high heterogeneity and the preliminary nature of the current evidence-base necessitate a conservative interpretation of their predictive power in routine clinical settings.

## Conclusion

In conclusion, advanced imaging biomarkers such as CC FD% and m-GCIPL thinning have been consistently associated with DR progression in longitudinal studies, although the current evidence is limited by low to very low certainty. These markers should be viewed as promising research tools rather than ready-for-routine clinical endpoints until standardized protocols and diverse population validations are achieved.

## Supplementary Information

Supplementary Material 1

## Data Availability

The datasets used and/or analyzed during the current study are available from the corresponding author on reasonable request.

## Abbreviations

DM: Diabetes mellitus
DR: Diabetic retinopathy
NPDR: Non-proliferative diabetic retinopathy
PDR: Proliferative DR
CFR: Color fundus photography
ETDRS: Early treatment diabetic retinopathy study
OCT: Optical coherence tomography
OCTA: OCT angiography
MeSH: Medical subject headings (MeSH)
HRs: Hazard ratios
ORs: Odds ratios
CIs: Confidence intervals
QUIPS: Quality in prognosis studies
GRADE: Certainty of evidence assessment
HbA1c: Glycemic control
GCIPL: Ganglion cell and inner plexiform layer
pRNFL: Peripapillary retinal nerve fiber layer
IZ: Length of the interdigitation zone
m-GCIPL: Macular GCIPL
AUC: Area under the curve
VD: Vascular density
MP: Macular perfusion
SVC: Superficial capillary plexus
ISI: Ischemic index
RDR: Referrable DR (RDR)
ERG: Electroretinogram
EPO: Erythropoietin
