# Supplementary material for: Prognostic value of advanced imaging biomarkers for the progression of diabetic retinopathy: a systematic review
Source: Diabetol Metab Syndr. 2026 Apr 28;18:133. doi: 10.1186/s13098-026-02159-3 (PMC13262439; doi:10.1186/s13098-026-02159-3)
Supplement: Supplementary file 1 — Supplementary Material 1 [file 13098_2026_2159_MOESM1_ESM.pdf]

Question: High flow deficit in the choriocapillaris (CC FD%) versus low flow deficit in the choriocapillaris (CC FD%) for Progression of Diabetic Retinopathy in Patients with Type 2 Diabetes.

Certainty assessment							№ of patients		Effect		Certainty	Importance
№ of studies	Study design	Risk of bias	Inconsistency	Indirectness	Imprecision	Other considerations	High flow deficit in the choriocapillaris (CC FD%)	[comparition} Low flow deficit in the choriocapillaris (CC FD%)	Relative (95% CI)	Absolute (95% CI)		
**Progression of Diabetic Retinopathy (CC FD%)**												
2	non-randomised studies	serious[a]	very serious[b]	not serious	very serious[c]	none		304/1849 (16.4%)	**RR 2.33** (1.13 to 4.84)	**219 more per 1.000** (from 21 more to 631 more)	⨁◯◯◯ Very low[a,b,c]	CRITICAL

CI: confidence interval; **RR:** risk ratio

Explanations

a. Certainty was downgraded by one level due to serious risk of bias. The body of evidence consists of only two studies, one classified as high risk (28) and the other as moderate risk (34).

b. Certainty was downgraded two levels for Very Serious Inconsistency. The meta-analysis revealed extremely high statistical heterogeneity (I^2 = 96.0%), driven by very different effect sizes between the two studies (RR 3.41 vs. RR 1.62).

c. Certainty has been downgraded by one level for Serious Imprecision. The 95% confidence interval of the pooled result (RR 2.33) is very wide (1.13 to 4.84), and its lower bound (1.13) is very close to the no-effect line (1.0), indicating substantial uncertainty.

Question: m-GCIPL damage versus no m-GCIPL damage for Progression of Diabetic Retinopathy in Patients with Type 2 Diabetes.

Certainty assessment							№ of patients		Effect		Certainty	Importance
№ of studies	Study design	Risk of bias	Inconsistency	Indirectness	Imprecision	Other considerations	(Intervention): m-GCIPL damage	(Comparison): No m-GCIPL damage	Relative (95% CI)	Absolute (95% CI)		
**m-GCIPL**												
2	non-randomised studies	serious[a]	not serious	serious[b]	not serious	none		138/472 (29.2%)	**HR 1.55** (1.28 to 1.87)	**123 more per 1.000** (from 65 more to 184 more)	⨁◯◯◯ Very low[a,b]	CRITICAL

CI: confidence interval; **HR:** hazard ratio

Explanations

a. Certainty was downgraded by one level for serious Risk of Bias. The body of evidence consists of two studies, one classified as low risk (32) and the other as moderate risk (37).

b. Certainty was downgraded one level to Indirect Evidence. The two studies were combined, but measured the biomarker by methodologically different methods (one measured the rate of thinning (32), the other the thickness at baseline (37).
